# Supplementary material for: Bayesian neural networks for stock price forecasting before and during COVID-19 pandemic
Source: PLoS One. 2021 Jul 1;16(7):e0253217. doi: 10.1371/journal.pone.0253217 (PMC8248663; doi:10.1371/journal.pone.0253217)
Supplement: Attachment — Submitted filename: ReviewPLOSoneBayes_stockmarket.pdf [file pone.0253217.s001.pdf]

Review Comments to the Author

Please use the space provided to explain your answers to the questions above. You may also include additional comments for the author, including concerns about dual publication, research ethics, or publication ethics. (Please upload your review as an attachment if it exceeds 20,000 characters)

The authors sincerely thank the Editor and Reviewers for valuable comments.

Reviewer #1: The abstract should be reformulated. This is a very important part of the article. The authors spend too much writing on background. Instead, the authors should better present their research, the main contributions and results, and the conclusions that might be drawn from these results.

The authors have rewritten major parts of the abstract to highlight the key contributions and significance of the paper.

In my opinion, the introduction is not well focused. Background should be brief. The originality (novelty) and relevancy of the study should be established with better efforts. My understanding is that this study is an empirical one. Thus, it is expected that the introduction should also include hypothesis and objectives of the study, followed by a justification of the methodology. The current manuscript needs much improvements on all these aspects.

The introduction has been organised, this is a methods paper and follows a style where the aim and goals are given in the introduction.

In the introduction, authors claim to study stock market prediction using Bayesian neural networks. However, from the data description, I see the study actually only considers 4 stocks, each from a different country though. Predicting the stock price of a particular company is a very different task from stock market prediction. I believe the authors need more careful wording, and should articulate their research question in the introduction.

The research aims and goals - along with research questions - is highlighted in the revised introduction.

More on the data, I don't see any explanation as to why these companies are selected for experiments. Are there any criteria? Also, how to decide the date after which the stock price is affected by COVID-19. Moreover, the authors choose different dates for different stocks/countries. Reasons and justifications are expected here.

The selected stocks are given due to geography and also due to the effect of various types of lockdowns during the course of the 2020 COVID-19 pandemic that affected the GDP. Although more datasets can be incorporated, we restrict our study to stocks from these countries which had diverse GDP forecasts during the pandemic. Moreover, one of the selected stocks feature COVID-19 mask manufacturing company, while others feature regarding luxury goods for a diverse stock-market analysis.

This is further highlighted in the introduction section of the paper.

The abbreviation FNN-Adam and FNN-SGD are used without mentioning full names. Please check if the use of other abbreviations is of the same problem.

These have been defined and introduced in the revision.

I think one of the main tasks of this study is to compare Bayesian neural network with other neural network methods in terms of forecasting performance. Is it sound to consider only one performance measure (RMSE) here?

The following has been given with past work cited where other forms of errors have been used.

"We use RMSE since it is one of the key performance measures for time series forecasting in the literature. Other measures such as Mean Absolute Error (MAE), and Normalised Mean Squared Error (NMSE) can also be used. Note that in our past work (Chandra and Zhang, 2012), we found that NMSE for example, does not change the key conclusions for time series problems, hence we used RMSE only."

From my perspective, the conclusion is quite weak. A more detailed conclusion is needed. The novel method applied here does not seem to outperform state-of-art machine learning methods, at least I can't see it from the conclusion part. The better performance prior COVID-19 is no surprise indeed, and thus does not add any weight to the conclusion part. I expect to see more intelligent conclusions such as the real advantages of this novel Bayesian neural network method. Probably, a comparison with other state-of-art studies would be helpful. This can also be added to the discussion part.

The authors agree and the conclusion has been rewritten to address the concerns of the reviewer.

English writing must be carefully revised. Usually, use of WE/OUR in the academic writing should be avoided. I have encountered many grammar mistakes and typos while reading. I list them as below but there are probably more in the manuscript. Thought I am not a native speaker, I feel like the manuscript would benefit from a proof read by a native speaker.

We use this style (we/our) for active voice in the paper which is common in machine learning and has been used in all my papers in past, published in leading machine learning journals and would like to keep it this way. We have reduced the usage in many sentences, especially in the conclusion section.

The manuscript has been checked and typos have been fixed including the following issues.

Mistakes I have spotted:

Line 16, "Markov Chain Monte Carlo (MCMC) methods provides a means…" fixed in the text

Line 17, "As the size of model and data continues increases…" fixed in the text

Line 205-206, "The probabilistic neural network model employs the posterior distribution to provides uncertainty quantification on the predictions." fixed in the text

Line 293, "…we set the burn-in rate is 0.5" fixed in the text

Line 376, "We good prediction accuracy is needed not just for the day ahead, …" fixed in the text

Line 430, "COVID-19 which is not surprising given internal market-crush" fixed in the text

Line 431, "…its is more challenging to provide forecasting during COVID-19…" fixed in the text

The authors sincerely thank the reviewer.

Reviewer #2: This paper applies a Bayesian neural networks for multi-step-ahead stock market forecasting before and during COVID-19. But in this version，I don't think the author has made it clear where their novelty ies, is it the novelty of method, or is it the novelty of predicting the stock price changes before and after covid-19? In the paper, the author mentioned that "In the literature, there has not much been done using Bayesian neural networks for stock markets that features robust uncertainty quantification. We can use them to harness power of neural networks that provides good prediction accuracy and also quantify uncertainty. Moreover, there has not been much work that shows how robust machine learning models such as neural networks perform post COVID-19 given major changes in the international stock market with disruptions in international trade and prediction." ，but actually in the listed or not listed references，there are some papers for forecasting in COVID-19. The authors should state exactly the difference of this paper from others, not in a general way. Besides, I list other questions for revision reference.

Yes there are many papers out there about COVID-19 forecasting with machine learning methods, but use of Bayeian neural networks is limited, especially in case of stock market prediction. The introduction has been edited to highlight the major differences.

The introduction has been edited to stress the novelty of the paper:

"The novelty lies in the use of Bayesian neural networks which provides better model uncertainty quantification when compared to classical neural networks."

1. Why Bayes neural network is suitable（even superior）for stock price forecasting. The highlights of this paper should be further addressed.

"In case of stock market prediction, uncertainty quantification has been a challenge and really important." This has been added to further stress importance of the method in introduction

The following has been added in the conclusion section: " Although machine learning methods provide accurate prediction, their applicability in stock market prediction remains due to volatility of the market and hence model validity is important. With robust uncertainty quantification via Bayesian inference, investors would find more confidence in predictions using Bayesian neural networks. "

The highlights have also been edited.

2. The authors choose 4 stock prices from 4 countries. But I think the selected stocks are not the most representative market stock. Why these datasets are selected should be further clarified.

The selected stocks are given due to geography and also due to the effect of various types of lockdowns during the course of the 2020 COVID-19 pandemic for the GDP. Although more datasets can be incorporated, we restrict our study to stocks from these countries which had different GDP forecasts during the pandemic. Moreover, one of the selected stocks feature COVID-19 mask manufacturing company, while others feature regarding luxury goods for a diverse stock-market analysis.

This is further highlighted in the intro section of the paper.

3. Two other methods are compared with the Bayes neural network, that is, FNN-Adam and FNN-SGD, whose full names should be given when they first appear. These have been defined and introduced in the revision.

4. The abstract is poorly written. The authors do not clearly show the highlights and significance of this work. The abstract has been rewritten to better highlight significance.

5. The parameter settings in the experiment are very important. I think the authors should give an illustration of parameters in different forecasting models.

Revised Section 3.2 provides all the information such as the neural network model topology and MCMC sampling parameters.

6. “”In Setup-2, we include parts of the data during COVID-19 in the training set with all the training data from Setup-1.” What does ”parts” mean here？ I suggest the authors to give an exact time period and data length to show them. Besides，what is the objective of Setup 2.

The above has been rephrased to the following providing more information:

“In Setup-2, we include parts of the data during COVID-19 in the training set with all the training data from Setup-1 where the exact dates are given in Table 4. In general, we appended Setup-1 with data from March and April, 2020 that covers the first phase of the pandemic in the respective countries that affected the stocks. The major reason for doing this is to ensure that the training dataset covers the stock-market trend during the pandemic.”

The authors agree and hence the sentence has been edited with further details.

The authors sincerely thank the reviewer.
