# Supplementary material for: Bayesian neural networks for stock price forecasting before and during COVID-19 pandemic
Source: PLoS One. 2021 Jul 1;16(7):e0253217. doi: 10.1371/journal.pone.0253217 (PMC8248663; doi:10.1371/journal.pone.0253217)
Supplement: Attachment — Submitted filename: finalreview.pdf [file pone.0253217.s002.pdf]

Reviewer #1: The revised manuscript has addressed all my comments. I only have one additional suggestion. In the study, the authors use the novel method to predict stock prices of 4 individual companies, rather than market indices. I think it is more appropriate to describe it as "stock price forecasting" than "stock market forecasting". Thus, I suggest to revise the title and relevant parts in the main text.

Thanks very much for your time and valuable comments. This change has been made and highlighted.

Reviewer #2: The authors have carefully revised paper accorrdingt to my comments and other reviewr's comment. I suggest an acceptance.

Thanks very much for your valuable time and comments.

The authors sincerely thank the reviewers and the managing editor.
